# Quantitative HPLC-MS analysis of nucleotide sugars in plant cells following off-line SPE sample preparation

**DOI:** 10.1007/s00216-014-7746-3

**Published:** 2014-03-18

**Authors:** Robert Behmüller, Ines C. Forstenlehner, Raimund Tenhaken, Christian G. Huber

**Affiliations:** 1 Department of Molecular Biology, Division of Chemistry and Bioanalytics, University of Salzburg, Hellbrunner Straße 34, 5020 Salzburg, Austria; 2 Department of Cell Biology, Division of Plant Physiology, University of Salzburg, Hellbrunner Straße 34, 5020 Salzburg, Austria

**Keywords:** UDP-sugars, *ugd2,3* mutant, Porous graphitic carbon, Liquid chromatography, Electrospray ionization mass spectrometry, Off-line solid-phase extraction, *Arabidopsis thaliana*

## Abstract

An analytical workflow was developed for the absolute quantification of uridine diphosphate (UDP)-sugars in plant material in order to compare their metabolism both in wild-type *Arabidopsis thaliana* and mutated plants (*ugd2,3*) possessing genetic alterations within the UDP-glucose dehydrogenase genes involved in UDP-sugar metabolism. UDP-sugars were extracted from fresh plant material by chloroform-methanol-water extraction and further purified by solid-phase extraction with a porous graphitic carbon adsorbent with extraction efficiencies between 80 ± 5 % and 90 ± 5 %. Quantitative determination of the UDP-sugars was accomplished through HPLC separation with a porous graphitic carbon column (Hypercarb^TM^) which was interfaced to electrospray ionization Orbitrap mass spectrometry. The problem of instable retention times due to redox processes on the stationary phase were circumvented by grounding of the column effluent and incorporation of a column regeneration procedure using acetonitrile-water containing 0.10 % trifluoroacetic acid. The method was calibrated using external calibration and UDP as internal standard. Calibration functions were approximated by first- or second-order regression analysis for concentrations spanning three orders of magnitude. Upon injecting sample volumes of 2.65 μL, the limits of detection for the UDP-sugars were in the 70 nmol L^−1^ range. Six different UDP-sugars, including UDP-glucose, UDP-galactose, UDP-arabinose, UDP-xylose, UDP-glucuronic acid, and UDP-galacturonic acid were found in concentrations of 0.4 to 38 μg/g plant material. Data evaluation by analysis of variance (ANOVA) revealed statistically significant differences in UDP-sugar concentrations between wild-type and mutant plants, which were found to conclusively mirror the impaired metabolic pathways in the mutant plants.

**Electronic supplementary material:**

The online version of this article (doi:10.1007/s00216-014-7746-3) contains supplementary material, which is available to authorized users.

**Figure:**
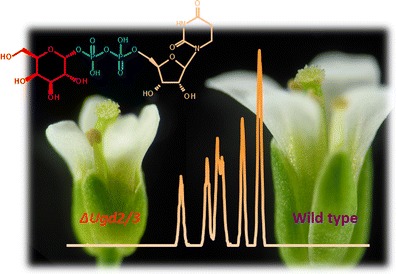
ᅟ

## Introduction

As nucleotide sugars in plants are responsible for the conversion of sun energy into usable plant biomass, research in this area is essential for a better understanding of the enzymatic pathways necessary for nucleotide sugar production [1]. Cellulose, a polysaccharide comprising a linear chain of several hundred to over ten thousand β(1 → 4) linked d-glucose units, is an important structural component of the primary cell walls of green plants. Cellulosic biomass is the biggest contributor to the production of biomass on the planet. It is synthesized at the plasma membrane using UDP-Glc as glycosyl donor, which represents the most prominent nucleotide sugar in plants and which is provided by either photosynthesis or from the cleavage of sucrose by sucrose synthase. Moreover, many trees contain high amounts of xylans, which are complex polysaccharides synthesized from UDP-xylose, and which account for up to 30 % of the wood biomass. Finally, the diversity of sugars found, e.g., in pectic polysaccharides clearly demonstrates the need for more than 10 different nucleotide sugars for biosynthesis [1–3]. The intercellular levels of nucleotide sugars as well as their corresponding glycosyltransferases have been shown to be of utmost importance to cell wall formation and characteristics.

**Fig. 1:**
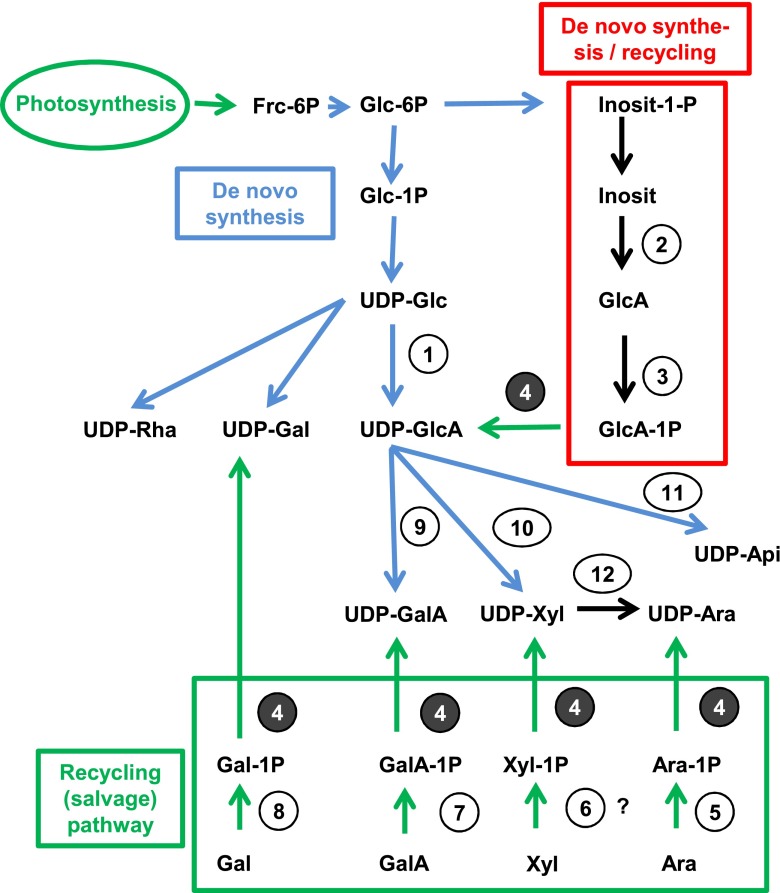
De novo synthesis and recycling pathways of UDP-sugars. Enzymes, *1* UDP-glucose dehydrogenase (UGD); *2* myo-inositol-oxygenase (MIOX); *3* glucuronic acid-1-kinase (Glucuronokinase); *4* UDP-sugar pyrophosphorylase (USP); *5* arabinose-1-kinase (Arabinokinase); *6* putative xylose-1-kinase (not known); *7* galacturonic acid-1-kinase; *8* galactose-1-P kinase (Galactokinase); *9* UDP-glucuronic acid 4-epimerase; *10* UDP-xylose synthase; *11* UDP-apiose synthase; *12* UDP-xylose epimerase; the “de novo synthesis/recycling” pathway including inositol-1-P, inositol, glucuronic acid, and glucuronic acid-1-P is also referred to as “Inositol oxygenation pathway”

There are two major routes for UDP and guanosine diphosphate (GDP)-sugar production: the de novo pathway as well as the salvage pathways (Fig. 1) [1]. The de novo pathway focuses on the interconversion of nucleotides to produce UDP and GDP sugars. Other than the de novo pathway, the salvage pathway produces nucleotide sugars by phosphorylation and subsequent pyrophosphorylation of monosaccharides, which are released by cell wall turnover or spontaneous hydrolysis of energy-rich nucleotide sugars in cells. The phosphorylation is done by sugar-specific monosaccharide-1-kinases, whereas the pyrophosphorylation is done by a common UDP-sugar pyrophosphorylase which accepts at least six different sugar-1-phosphates as substrate (Glc-1-P, Gal-1-P, GlcA-1-P, GalA-1-P, Ara-1-P; Xyl-1-P [1]). Fucose recycling occurs via a dual functional kinase/pyrophosphorylase enzyme [4]. Although UDP-sugars are being recycled via the salvage pathway, the major part of these sugars is produced via the de novo pathway as was demonstrated by radioactive precursor feeding to *Arabidopsis* cell cultures (Fig. 1) [2].

The enzyme UDP-glucose dehydrogenase (UGD) oxidizes UDP-Glc into UDP-GlcA, a common precursor for the UDP-sugars of galacturonic acid, xylose, arabinose, and apiose. UDP-GlcA is thus the main precursor for the synthesis of hemicelluloses and pectic polymers, providing roughly 50 % of the cell wall biomass of *Arabidopsis* leaves. Despite the importance of UDP-GlcA for plant biomass, almost no data is available about the absolute concentration of this metabolite in plant cells. An *Arabidopsis* double mutant, which has two of the four genes for UGD synthesis (*ugd2,3* [5, 6]) knocked out, was shown to have only 40 % of the wild-type activity of UGD but exhibits profound changes in the cell wall composition. This suggests that the *ugd2,3* mutant is compromised in some of the nucleotide sugars, limiting the availability of them for glycosyltransferases [5, 6]. Several reports have been published demonstrating the analysis of nucleotide sugars. In general, the most versatile and sensitive detection method for cellular metabolites is mass spectrometry (MS) [7]. Although the combination of gas chromatography (GC) with MS represents a powerful tool for carbohydrate research, large and thermolabile compounds such as nucleotide sugars, more specifically UDP-sugars, or large oligosaccharides cannot be analyzed by GC-MS because of their limited volatility and instability during derivatization. Therefore, liquid chromatography remains the method of choice when dealing with highly polar compounds like UDP-sugars. One option for separating highly polar and charged compounds is ion exchange chromatography, which is, however, not compatible with MS due to the use of salts solutions for elution [7].

Rabina et al. reported successful HPLC separation and analysis of nucleotide sugars, including UDP-sugars using ion-pair reversed-phase HPLC with a Discovery C18 octadecylsilica column from Supelco and matrix-assisted laser desorption/ionization time-of-flight mass spectrometry [8]. They published HPLC separation of UDP-sugars, in which baseline resolution was not achievable for UDP-Gal and UDP-Glc [8]. Pabst et al. successfully separated and analyzed 35 nucleotide sugars, including UDP-sugars, by employing porous graphitic carbon (PGC) chromatography on a Hypercarb column. Nonetheless, they reported problems of retention time instabilities due to the nature of the PGC material [9]. Alonso et al. published data in which they used high-performance anion-exchange chromatography on an Ionpac AS11 column to separate UDP-sugars [10], while detection was done using a triple-quadrupole mass spectrometer. Due to the high concentration of NaOH in the eluent, an anion suppressor was needed [10]. They were able to separate isobaric UDP-sugars like UDP-GalA, UDP-GlcA, UDP-Gal, UDP-Glc, UDP-Ara, and UDP-Xyl.

Based on the requirement to discriminate isobaric sugars and to sensitively detect UDP-sugars by electrospray ionization mass spectrometry (ESI-MS), in this work we aimed at developing a PGC-based chromatographic system for the separation of UDP-sugars, which is stable and suitable for subsequent analyte detection by ESI-MS. Method development has to overcome the instability of chromatographic retention [10–12], which is believed to be a consequence of redox reactions involving the stationary phase due to applied electrospray voltage [12, 13], and to allow UDP-sugar detection in plants in physiologically relevant concentrations. Finally, we want to demonstrate the applicability of our method by comparing the absolute UDP-sugar concentration in wild-type *Arabidopsis thaliana* leaves as well as in the *ugd2,3* double mutant. By analyzing the *UGD* double mutant, we expect a significant decrease of UDP-sugars downstream of UGD. However, results may vary depending on the amount of UDP-sugars produced by the salvage pathway and the inositol pathway because it was shown for *ugd2,3* that the severe seedling phenotype could be partially rescued by the inositol pathway [5].

## Materials and methods

### Chemicals and reagents

Deionized water was prepared by a Milli-Q System (Millipore Corporation, Billerica, USA). HPLC-grade acetonitrile was purchased from Sigma-Aldrich (Steinheim, Germany). Standards of uridine 5′-diphosphate disodium salt hydrate (UDP, ≥96.0 %), uridine 5′-diphosphate-glucose (UDP-Glc), uridine 5′-diphosphate-galactose (UDP-Gal), uridine 5′-diphosphate-glucuronic acid (UDP-GlcA), were purchased from Sigma-Aldrich (Steinheim, Germany). Uridine 5′-diphosphate-arabinose (UDP-Ara), uridine 5′-diphosphate-xylose (UDP-Xyl) and uridine 5′-diphosphate-galacturonic acid (UDP-GalA) were from Carbosource (University of Georgia, Athens, GA, USA). UDP-sugars were quantified by UV absorbance on a Nanodrop ND-1000 spectrophotometer (NanoDrop Products, Thermo Scientific, Wilmington, DE, USA), using UDP as reference. Trifluoroacetic acid (≥99.5 %), HPLC grade methanol, and 2-propanol were purchased from Fluka Analytical (Buchs, Switzerland). Ammonia (25 %) and chloroform (≥99.0–99.4 %) were purchased from Merck (Darmstadt, Germany). Peek capillaries of various sizes as well as nuts, ferrules, sleeves, finger tights, and metal unions were purchased from Upchurch Scientific (Oak Harbor, USA). Supelclean ENVI-Carb SPE Tubes (3 ml, 0.25 g, particle size: 120–400 mesh) were obtained from Sigma-Aldrich. Sample vials, vial inlets, and vial snapcaps were purchased from VWR International (Radnor, USA).

### Plant material and growth conditions

*A. thaliana* seeds, ecotype *Columbia* (wild type) and *ugd2,3* were grown in standard fertilized soil (type ED73) in a growth chamber at 23 °C with 8 h light and 16 h dark period. During the dark phase, temperature was decreased to 18 °C. Humidity was set to 60 % during cultivation. Samples were taken from 4-week-old *A. thaliana* wild-type and *ugd2,3* mutant plants. Four biological replicates were grown from each species and three samples were picked from each biological replicate. One sample consisted of two medium-sized leaves that were put into pre-weighted reaction tubes. To facilitate the pulverization under liquid nitrogen, a 3-mm tungsten carbide ball was put into the tubes before sample collection. After harvesting the plant material, sample tubes were weighted and immediately stored in a liquid nitrogen reservoir to facilitate immediate cooling. The plant material was pulverized in liquid nitrogen-cooled Teflon carriers for 2 min using a ball mill (Model PM200, Retsch, Düsseldorf, Germany). The plant material was stored at −80 °C after harvesting and pulverization.

### Liquid-liquid extraction and solid-phase extraction procedure

Liquid-liquid extraction according to Lunn et al. [11] was applied to extract the UDP-sugars from the plant material. Briefly, 250 μL of the quenching solution, consisting of chloroform and methanol in a 3:7 (*v*/*v*) ratio, were added to the pulverized samples. The samples were incubated at −20 °C for 2.0 h and vigorously mixed every 30 min. UDP-sugars were extracted by adding 400 μL of water, followed by thorough vortexing and centrifuging the mixture at 14,000 rpm for 5.0 min. The aqueous layer was removed and stored. Afterwards, another 400 μL portion of water was added to the organic layer followed by another centrifugation step after which the aqueous layer was removed again and stored. The stored aqueous phases containing the UDP-sugars were vaporized using a vacuum concentrator (Model Concentrator plus, Eppendorf, Wesseling-Berzdorf, Germany) at 45 °C using the AL setting, which is recommended for alcoholic solutions. After vaporization, all samples of each biological replicate were re-constituted in water and the two extracts per sample were combined and diluted with water to a total volume of 1 mL.

Solid-phase extraction (SPE) as described by Pabst et al. [9] was applied to further reduce sample complexity. The solid-phase extraction was performed with Supelclean ENVI-Carb SPE Tubes (3.0 mL, 0.25 g, particle size: 120–400 mesh) consisting of graphitized non-porous carbon. The SPE columns were conditioned by flushing with 3.0 mL of 60 % acetonitrile in water containing 0.30 % formic acid adjusted to pH 9.0 with ammonia and then flushing with 3.0 mL of water. After sample application, washing steps were performed, first with 3.0 mL of water and afterwards with 1.0 mL of 60 % acetonitrile in water. Sample elution was done by perfusing the column with 2.0 mL of 60 % acetonitrile in water containing 0.30 % formic acid adjusted to pH 9.0 with ammonia. After elution of the sample from the SPE columns, a concentration step was performed by vaporizing the solvents and re-dissolving the samples in 250 μL of water.

Recoveries of liquid-liquid extraction and solid-phase extraction were determined individually for each UDP-sugar by extracting an aliquot of the respective UDP-sugar solution (10 μmol L^−1^) as described above and comparing the peak area obtained from triplicate HPLC analysis of the extracted solution with that of a standard solution having the same concentration. The peak areas were measured using an analytical HPLC system (Model U3000, Thermo Fisher Scientific, Germering, Germany) operated at a wavelength of 262 nm. The separation was performed at 25 °C on a Nucleosil 4000-7 PEI column (125 × 4 mm, particle size 7 μm) from Macherey-Nagel (Düren, Germany). The mobile phase consisted of solvent A: 2.5 mmol L^−1^ Tris adjusted to pH 7.2 with phosphoric acid; and solvent B: 2.5 mmol L^−1^ Tris with 1.5 M KCl adjusted to pH 8.0 with phosphoric acid. The gradient program started with 5.0 % solvent B which was increased to 95 % B within 5.0 min, afterwards, the gradient increased to 100 % B within 1.0 min, was held for 2.0 min and then, the gradient was stepped to 5.0 % B within 1.0 min and the column was re-equilibrated at 5.0 % B for 5.0 min. The flow rate was set to 1.3 mL min^−1^ with an injection volume of 20 μL operated with partial loop injection. A slightly different gradient program was used for the determination of SPE extraction efficiencies: starting conditions were 5.0 % solvent B, which was kept for 2.0 min. Then, solvent B was increased to 95 % in 3.0 min, afterwards, the gradient was increased to 100 % B within 1.0 min, which was kept for 2.0 min and then, the gradient was stepped to 5.0 % B within 1.0 min and the column was re-equilibrated at 5.0 % B for 5.0 min.

### High-performance liquid chromatography–mass spectrometry (HPLC-MS)

HPLC separations were performed on an UHPLC system (Model Accela, Thermo Fisher Scientific, Palo Alto, CA, USA) consisting of an Accela 1250 pump, an 80-Hz diode array detector (PDA) and an LC PAL DLW Option Autosampler with a 100 μL syringe (from CTC Analytics AG, Zwingen, Switzerland). A Hypercarb column (150 × 1 mm i.d., 5 μm particle size) was purchased from Thermo Fisher Scientific (Waltham, USA). The injection mode in all measurements was full loop injection using a metal loop of 2.65 μL volume. The syringe cleaning procedure comprised a washing step before and after sample injection, using methanol as washing solution. During measurements, the column oven was set to 30 °C and samples were stored at +4 °C.

The mobile phase consisted of solvent A, pure water, and solvent B, water with 0.30 % (*v*/*v*) formic acid adjusted to pH 9.0 with ammonia. Solvent C was acetonitrile, and solvent D consisted of 80 % (*v*/*v*) acetonitrile-water containing 0.10 % (*v*/*v*) trifluoroacetic acid. Before starting analytical measurements, a column regeneration procedure was performed to establish a defined redox state of the Hypercarb PGC stationary phase, which is required to achieve stable elution and separation of highly polar compounds [14]. The column regeneration method consisted of 180 min flushing with solvent D. Afterwards, re-equilibration was performed by rinsing for 90 min with 53 % A, 40 % B, and 7.0 % C. This method was used as a sequence “primer”, meaning that each sequence of measurements started with this column regeneration phase. It was also observed that storing the column in 53 % A, 40 % B, and 7.0 % C was vital to ensure stable retention times. Column regeneration was conducted at a flow of 100 μL min^−1^ which resulted in a back pressure of 135 to 160 bar, depending on the redox state of the column. Sample measurements were performed with a gradient program that employed the starting conditions of 53 % A, 40 % B, and 7.0 % C. Within 15 min, a linear gradient to 50 % A, 32.5 % B, and 17.5 % C was programmed. Subsequently, a composition of 5.0 % A, 5.0 % B, and 90 % C was adjusted within 0.10 min and kept for 6.9 min. From 21 to 21.1 min, starting conditions of 53 % A, 40 % B, and 7.0 % C were restored and kept for 8.9 min (30 min total run time). Before starting the next chromatographic run, a regeneration phase of 20 min flushing with 100 % D, followed by 90 min flushing at starting conditions (53 % A, 40 % B, and 7.0 % C) was performed to improve retention time stability.

Mass spectrometric measurements were carried out on an Orbitrap mass spectrometer (Model Exactive, Thermo Fisher Scientific, Bremen, Germany). UDP sugars were detected in negative mode as deprotonated molecules. Fine tuning of the mass spectrometer was done by hyphenating the HPLC system to the IonMAX ESI source operated under starting conditions and post-column addition of a 5.0 μL min^−1^ flow of 100 μmol L^−1^ UDP-Glc standard solution through a T-connector between the HPLC system and the mass spectrometer. The tuning at *m*/*z* 565 was conducted manually as well as automatically by the Xcalibur software. The following scan parameters were applied during measurements: scan range, *m*/*z* 100 to 2,000 ; resolution, 100,000; microscans, 1; AGC target, 1e6; maximum inject time, 50 ms. Source settings had to be established for a flow rate of 100 μL min^−1^ with a spray voltage of 1.6 kV, capillary temperature of 200 °C, sheath gas of 35 arbitrary units and aux gas at 15 arbitrary units. Lock masses were used for a formic acid dimer ([M_2_ + Na-2H]^−^, *m*/*z* 112.98563) and trifluoroacetic acid dimer ([M_2_-H]^−^, *m*/*z* 226.97845), which were permanently present during the measurements. Fragmentation experiments were performed on a linear ion trap–Orbitrap mass spectrometer (Model LTQ Orbitrap XL, Thermo Fisher Scientific).

## Results and discussion

### Establishing stable retention times for UDP-sugars on PGC

**Fig. 2:**
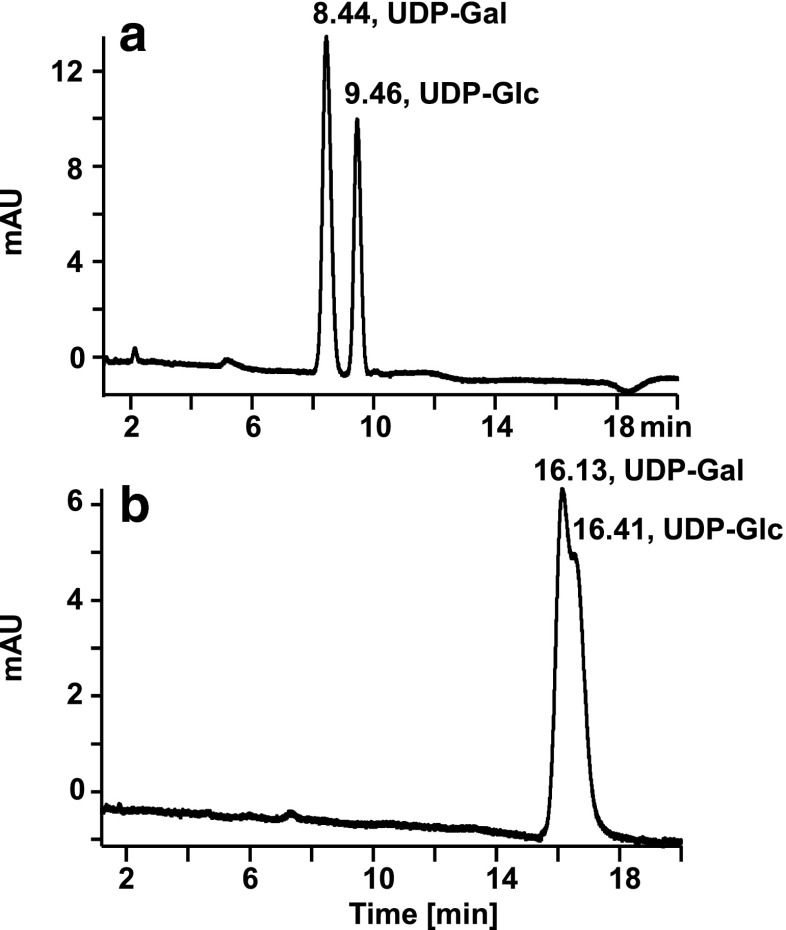
Influence of the potential applied to the ESI source on retention of UDP-sugars. Chromatogram obtained with a newly installed or regenerated PGC column (**a**); chromatogram obtained after 20-min utilization of the column connected to the ESI source of an Orbitrap mass spectrometer (**b**). Conditions: column regeneration was performed with 80 % acetonitrile in water containing 0.10 % trifluoroacetic acid for 20 min, the sample measurements were done with the gradient program described in the materials and methods section for HPLC-MS; detection, UV, 262 nm; sample. UDP-Gal (100 μmol L^−1^) and UDP-Glc (100 μmol L^−1^)

Figure 2 illustrates the effect of the electrical potential applied to the ESI source on chromatographic retention of UDP-sugars on PGC. While UDP-Gal and UDP-Glc were readily separated to baseline using a freshly installed or regenerated column (Fig. 2a), the separation rapidly deteriorated after the first run showing almost coelution of the two UDP-sugars at higher retention times (Fig. 2b). In a third run, both compounds coeluted at a retention time of approximately 18 min (chromatogram not shown). We believe that oxidation due to the oxidative potential applied to the ESI source makes the stationary phase more hydrophilic which entails increased retention of the highly hydrophilic UDP-sugars. Unfortunately, this increased hydrophilicity goes along with an almost complete loss of selectivity towards the different UDP-sugars. Selectivity and shorter retention could be fully restored upon extensive column rinsing with 80 % (*v*/*v*) acetonitrile containing 0.10 % (*v*/*v*) trifluoroacetic acid for 3 h, leading to a chromatogram equivalent to that shown in Fig. 2a. Since the oxidative potential applied to the ESI source is known to have an influence on the redox state of the PGC stationary phase, electrical grounding of the column effluent was performed in order to stabilize retention times. Several ways of grounding of the chromatographic column were tested, such as grounding the stainless steel housing of the column directly by attaching grounded metal clamps to it (data not shown). Nevertheless, the most efficient way of grounding was found by introducing a grounded gold electrode into a T-connector (M-540 from Upchurch, Oak Harbor, WA, USA), that was mounted between the column outlet and the inlet into the ESI source using two short pieces of 100 μm i.d. fused silica capillaries.

Even after proper grounding of the column effluent, retention times were still considerably unstable especially during long sequences of measurements. In consequence, retention time stability was further increased by performing a 20 min column regeneration step after each run before the column was equilibrated to starting conditions for 90 min. Through using such a 140 min gradient, regeneration, and re-equilibration program, it was possible to achieve sufficiently stable retention times over 72 h of constant operation, such that baseline separation of all isobaric UDP-sugar pairs was given in all measurements and also their elution order remained unchanged, which facilitated the unequivocal identification of the separated UDP-sugars.

### Separation and qualitative analysis of UDP-sugars

**Fig. 3:**
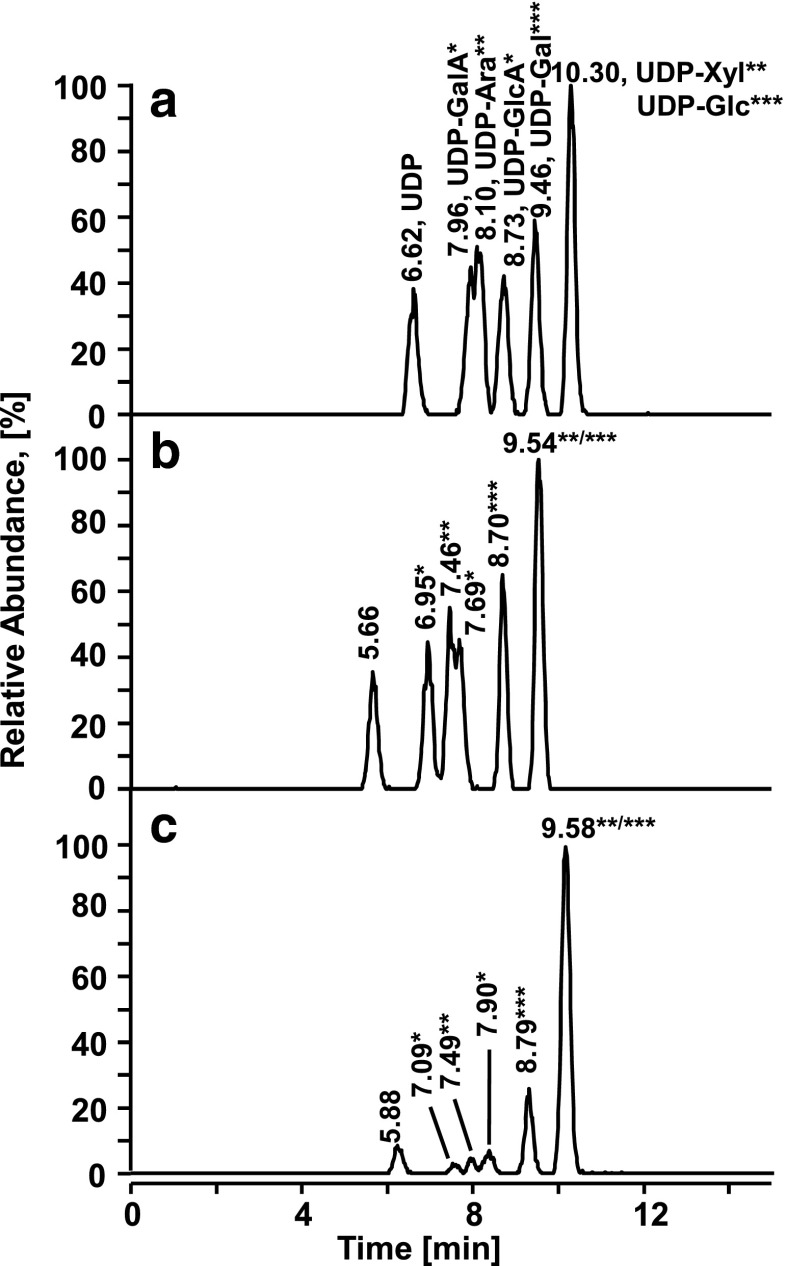
Separation of UDP and six UDP-sugars at the beginning (**a**), after 60 h (approx. 25 runs) of a measurement sequence (**b**), and (**c**) in a real sample extracted from *Arabidopsis* leaves. Chromatographic conditions are given in the materials and methods section; sample, 1.0 μmol L^−1^ each compound, 2.65 μL injected; extracted ion current chromatograms (EICC) were obtained from the raw data at the respective monoisotopic masses of the deprotonated UDP-sugars (402.9949 Da for UDP, 535.0371 Da for UDP-Xyl and UDP-Ara, 565.0477 Da for UDP-Gal and UDP-Glc, 579.0270 Da for UDP-GalA and UDP-GlcA) with a mass window of 10 ppm and five-point Gaussian smoothing. Pairs of isobaric UDP-sugars are labeled with *asterisks*

Since some of the measured UDP-sugars are isobaric and cannot be distinguished in a conventional mass spectrometric measurement, they have to be differentiated either based on specific diagnostic fragments or based on their chromatographic retention times. Fragmentation was carried out on all UDP-sugar standards seeking for specific fragments that would indicate the certain UDP-sugar. Unfortunately, all fragments observed could only be assigned to the nucleoside and nucleotide phosphates but the sugar itself was lost as neutral loss and therefore no differentiation based on fragmentation could be achieved (data not shown). The separation of UDP and six UDP-sugars of interest for this study is illustrated in Fig. 3. To get an impression of the stability of the system, chromatograms are shown from the beginning (Fig. 3a) and after 60 h (Fig. 3b) of a series involving the measurements of calibration standards and real samples. It can be seen that retention times decreased, however, the separation pattern was fully retained which facilitated the unambiguous assignment of the eluting compounds. Original retention could readily be restored by applying the column regeneration procedure described above, which was generally employed after completing a series of calibration and real sample measurements taking about 72 h. Figure 3 clearly demonstrates that the pairs of isobaric UDP-sugars, UDP-GalA/UDP-GlcA and UDP-Ara/UDP-Xyl are sufficiently separated to allow their unequivocal identification, while UDP-GalA and UDP-Ara are only partially separated and UDP-Xyl and UDP-Glc are coeluting but have different molecular masses.

### Liquid-liquid extraction and solid-phase extraction

**Fig. 4:**
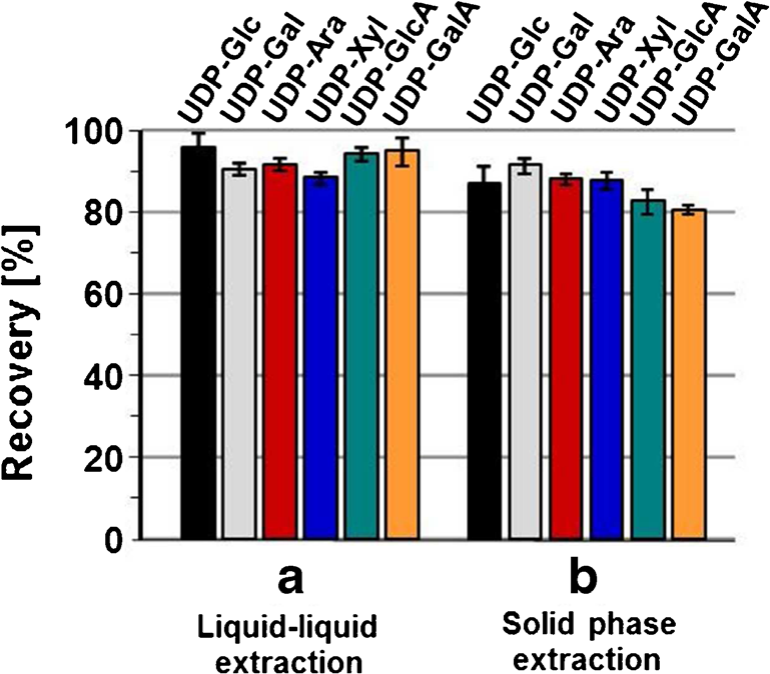
Analyte recoveries after liquid-liquid extraction (**a**) and solid-phase extraction (**b**). Recoveries were calculated from triplicate HPLC analysis of three to five replicate extractions of 10 μmol L^−1^ standard solution of the respective UDP-sugars. The diagram shows arithmetic averages and relative standard deviations of all analyses performed; sample size in (**a**): *N* = 9 for UDP-Glc, UDP-Xyl, UDP-GalA, and UDP-GlcA, *N* = 15 for UDP-Ara and UDP-Gal, sample size in (**b**): *N* = 31 for UDP-Glc, *N* = 15 for UDP-Ara, UDP-Xyl, UDP-Gal, UDP-GlcA, UDP-GalA

Due to the lack of isotope-labeled standards for the UDP-sugars, recoveries of the two liquid-liquid- and solid-phase extraction steps had to be determined by separate experiments through comparing analyte responses from untreated standard solutions with those obtained upon employing the respective extraction procedures. In order to obtain reliable values for the recoveries, at least ten extraction experiments were performed, and the results are summarized in Fig. 4. Measured recoveries were generally higher than 80 % proving that the analytes can be efficiently extracted with water and subsequently enriched on porous graphitic carbon material. The relative standard deviations for the extraction recoveries were all below 5 %, ranging between 1.4 and 3.9 % for liquid-liquid extraction (Fig. 4a) and 1.3 and 4.3 % for solid-phase extraction (Fig. 4b), which clearly demonstrates that the analytes can be reproducibly extracted and enriched from aqueous solution.

### Quantitative analysis of UDP sugars

External calibration was realized by triplicate measurements of standard solutions containing all nucleotide sugar standards in concentrations ranging from 0.010-10 μmol L^−1^. Peak areas were determined in extracted ion current chromatograms by applying the *Genesis* peak integration algorithm integrated in Xcalibur 2.1 with a mass tolerance window of 10 ppm as well as a *Gaussian* peak smoothing of 5 points (for representative chromatograms, see Fig. S1, Electronic Supplementary Material). Peak areas from these measurements were normalized using the average peak area of UDP, which was added as internal standard at 1.0 μmol L^−1^.

Parameters for calibration curves for each of the UDP-sugars are collected in Table 1. All calibrations were well modeled by linear regression over at least 2 orders of magnitude except for the calibration of UDP-galactose, which was better approximated with a second order regression line (calibration graphs are available Electronic Supplementary Material, Fig. S2). Since the UDP-sugar concentrations in our plant samples for UDP-Ara, UDP-Xyl, UDP-GalA, and UDP-GlcA are much lower than those for UDP-Gal and UDP-Glc, we restricted the calibration range for the former analytes to 0.010–2.5 μmol L^−1^, while a concentration range of 0.19–10 μmol L^−1^ was calibrated for the latter. Visual inspection of the calibration for UDP-Glc also suggests a better fit by a quadratic function; however, due to the loss of one degree of freedom in the quadratic regression, confidence intervals for expectation values were not significantly improved. Therefore, we utilized the linear regression function for UDP-Glc. The determination of limits of detection based on signal-to-noise ratios was challenging because no noise was determinable in the ion current chromatograms extracted with ±10 ppm mass windows. Instead, we measured the lowest concentrations of UDP-sugars that yielded an unequivocal peak in the extracted ion current chromatograms. Concentration-based limits of detection were at about 70 nmol L^−1^ for all UDP-sugars, which were well below the concentrations that were expected to be relevant for the plant extracts.

**Table 1:** Parameters of the regression equations used for calibration of six UDP-sugars

	Parameters for linear or quadratic regression^a^			
Compound	*a*	*b*	*c*	*R* ^2^
UDP-Ara	0	135,512	7,677.6	0.9941
UDP-Xyl	0	152,814	7,307.8	0.9888
UDP-GalA	0	136,180	7,096.4	0.9886
UDP-Gal	−5,530.2	177,542	13,157	0.9960
UDP-Glc	0	91,908	29,314	0.9763
UDP-GlcA	0	139,121	6,810.7	0.9880

^a^Calibration equations fulfilling linear (first order) or quadratic (second order) regression analysis according to the Equation *A* = *ax* ^2^ + *bx* + *c*, *A* peak area, *x* analyte concentration

### Real sample measurements

**Fig. 5:**
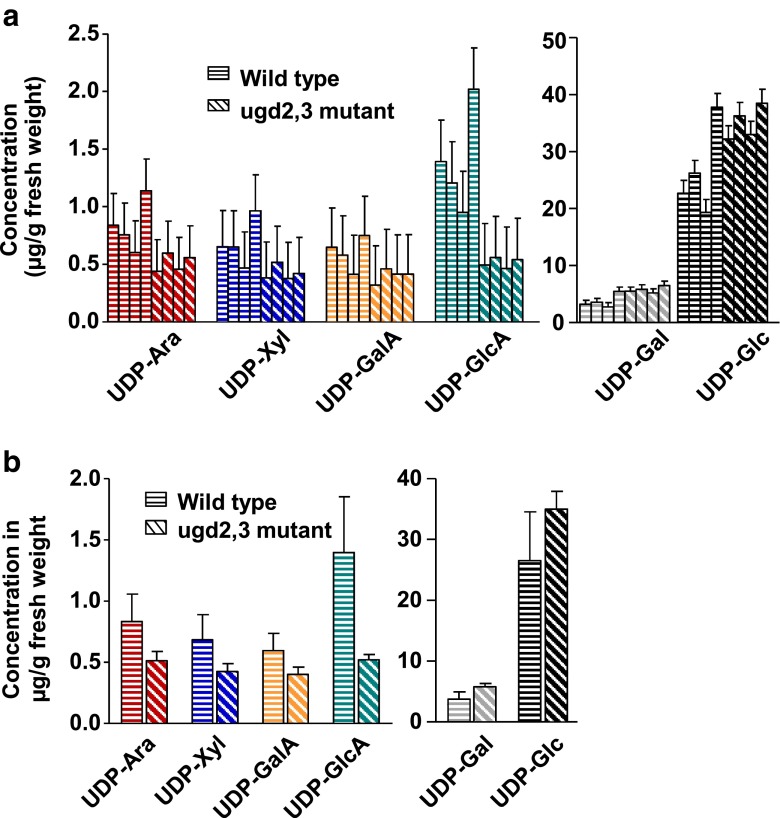
Comparison of UDP-Ara, UDP-Xyl, UDP-GalA, UDP-GlcA, UDP-Gal and UDP-Glc concentrations in *Arabidopsis* wild-type and *ugd2,3* mutant plants. The values shown are the results of triplicate measurement of four biological replicates each (**a**) or the averages over all four biological replicates (**b**). *Error bars* in **a** are reflecting the 95 % confidence intervals using linear or quadratic regression calculation. The *error bars* in **b** stand for the standard deviations calculated from the four biological replicates

After setup and optimization of the quantification method for UDP-sugars and preliminary measurements to estimate the concentrations of the UDP-sugars in the real sample, a comparison of UDP-sugar concentrations between wild-type and mutant *Arabidopsis* plants was feasible. Figure 3c exemplifies an extracted ion current chromatogram of the six analytes extracted from *Arabidopsis* wild-type leaves. The measured UDP-sugar concentrations and their corresponding confidence intervals are collected in Fig. 5a (a table listing the concentration values together with the corresponding confidence intervals is provided in the Electronic Supplementary Material, Table S1). It can be seen that UDP-Gal and UDP-Glc concentrations are approximately one order of magnitude higher than the concentrations of the other UDP-sugars. Our data shows that the UGD knockdown has a significant effect on UDP sugar concentrations that lie downstream of UGD (Fig. 5a and b). Corresponding data that is shown in Fig. 5a can be found in Table S1, Electronic Supplementary Material.

The relatively high relative standard deviations (RSD) that we encountered for the UDP-sugar concentrations, especially for the wild-type measurements, are primarily due to varying results coming from the four different biological replicates. The highest RSD for the wild-type biological replicates was found for the UDP-GlcA with a value of 32.7 %, the lowest RSD was that of UDP-GalA with a value of 23.7 %. The *ugd2,3* mutant showed an RSD range of 15.5 % as highest value for UDP-Xyl, and 8.2 % as the lowest value for UDP-GlcA. Similar values have also been reported by Josefczuk et al. [15], describing metabolic measurements done with biological replicates of *E. coli* which yielded RSDs of 19.5–27.1 %. According to Weckwerth et al. the relative technical standard deviation for GC/TOF measurements is around 10 %, for *Arabidopsis* however, samples can show “up to several fold variances” in metabolite levels [16]. This high biological variability makes it hard to reveal differences with statistical reliability [17, 18].

In order to reveal statistically significant differences in UDP-sugar concentrations between the four biological replicates each of wild-type and mutant plants, we decided to apply single-factor analysis of variance (ANOVA) [19], which can be used to test whether the sample means differ enough from each other to be caused by random error or not. The results clearly demonstrate that the concentrations of UDP-Ara, UDP-Gal, UDP-GalA, and UDP-GlcA differed significantly between the extracts obtained from wild-type and mutant plants, respectively (*p* values between 0.01 and 0.05). Only the concentrations of UDP-Glc and UDP-Xyl were found not to be significantly different (ANOVA results are provided in the Electronic Supplementary Material, Table S2). This result is also clearly corroborated in a comparison of the concentrations averaged over the four biological replicates (Fig. 5b), which demonstrates that the concentrations of UDP-Ara, UDP-GalA, and UDP-GlcA are reduced in the *ugd2,3* mutant, while the concentrations of UDP-Gal, UDP-Xyl, and UDP-Glc are increased or not significantly changed (Fig. 5b).

### Biological implications

The *Arabidopsis ugd2,3* mutant is characterized by a knockout in two of the four genes for UDP-glucose dehydrogenase (enzyme 1 in Fig. 1) resulting in a lower total enzyme activity for UGD of about 40 % relative to the wild type [5]. Our results clearly show a significant impact of knockout of *UGD* genes on the cellular concentrations of some UDP-sugars, especially UDP-GlcA, UDP-GalA, UDP-Xyl and UDP-Ara. UDP-sugars act as glycosyl donors for the synthesis of cell wall specific matrix polysaccharides. By altering the pathways leading to these UDP-sugars we would expect different cell wall composition [3, 5, 20]. Indeed cell wall composition analysis of *ugd2,3* showed a 25 % reduction in arabinose, xylose, and galacturonic acid [5]. Our data shows decreased UDP-sugar products that lie downstream of UGD (enzyme 1 in Fig. 1), such as UDP-GlcA, UDP-GalA, UDP-Xyl, and UDP-Ara. This is especially significant with the direct product of UGD, UDP-GlcA (Fig. 5a, b).

The regulation of the metabolite UDP-GlcA by UGDs was debated in the past. One scenario favors a strong feedback inhibition of UGDs by UDP-Xyl leading to a stable metabolite concentration which is only weakly influenced by moderate changes of the total UGD activity in a cell, because of the self-stabilizing feedback mechanism. This model was already questioned by Klinghammer et al. [21] based on kinetic measurements with recombinant *Arabidopsis* UGD enzymes, which showed only weak inhibition of Arabidopsis UGDs by UDP-Xyl. Moreover, the data provided in this study shows a direct correlation of UGD activity with the concentration of UDP-GlcA. Therefore, the total UGD activity directly controls the amount of UDP-GlcA whereas the influence of UDP-Xyl is negligible. Since the oxidation of UDP-Glc into UDP-GlcA is downregulated, an increase in UDP-Glc and UDP-Gal is expected and can be seen in our data (Fig. 5b). This results in increased galactan side chains of pectic polymers as reported by Reboul et al. [5] confirming that changes in nucleotide sugar concentrations have a great impact on polymer synthases.

## Conclusions

High-performance liquid chromatography on porous graphitic carbon remains one of the most potent methods for the chromatographic separation of sugars or sugar-derivatives. Nevertheless, the graphitic stationary phase is prone to changes in retention properties due to redox processes, which makes the method difficult to interface to mass spectrometry via electrospray ionization. Therefore, significant effort and time had to be put into developing a robust and stable method for separating and detecting highly polar UDP-sugars. We were able to implement a reliable workflow for the extraction, separation, and quantification of UDP-sugars in plant material. Absolute concentrations of these important plant metabolites can be reported to be in a range of 0.4–38 μg per gram fresh plant material. The repeatability of biological and technical replicates is sufficient to detect statistically significant differences in UDP-sugar concentrations between *A. thaliana* wild-type and *ugd2,3* mutant plants. The observed changes in UDP-sugar concentrations in knockout plants clearly corroborate the proposed biological function and importance of the enzyme UGD for several nucleotide sugars needed to synthesize plant cell walls.

## Electronic supplementary material

Below is the link to the electronic supplementary material.

ESM 1 (PDF 195 kb)
