# Supplementary material for: Quantitative HPLC-MS analysis of nucleotide sugars in plant cells following off-line SPE sample preparation
Source: Anal Bioanal Chem. 2014 Mar 18;406(13):3229–37. doi: 10.1007/s00216-014-7746-3 (PMC3992224; doi:10.1007/s00216-014-7746-3)
Supplement: Supplementary file 1 — (PDF 195 kb) [file 216_2014_7746_MOESM1_ESM.pdf]

Analytical and Bioanalytical Chemistry

Electronic Supplementary Material

Quantitative HPLC-MS analysis of nucleotide sugars in plant cells following off-line SPE sample preparation

Robert Behmüller, Ines C. Forstenlehner, Raimund Tenhaken, Christian G. Huber

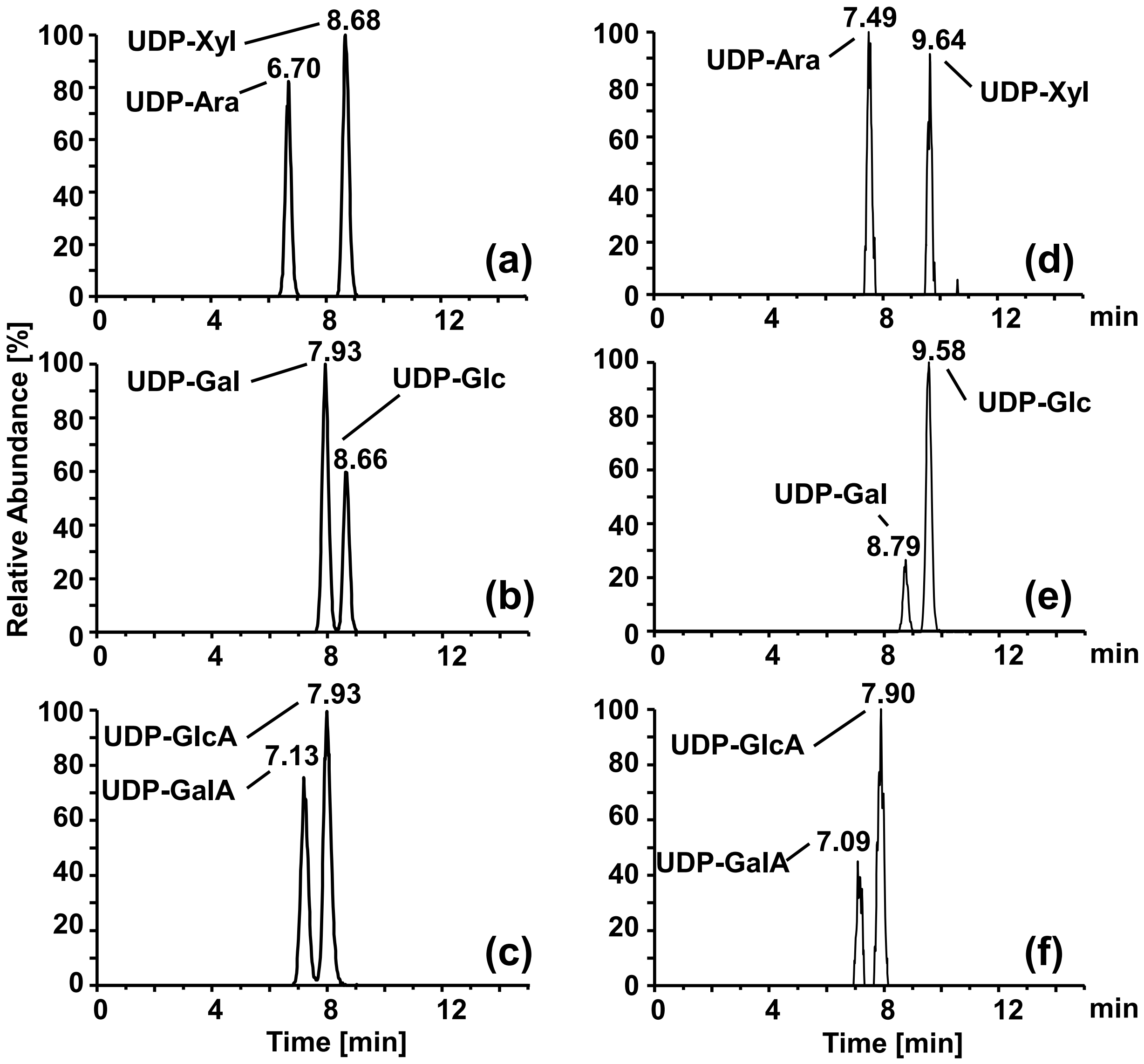

Fig. S1. Extracted ion current chromatograms (EICC) of UDP-Xyl and UDP-Ara (m/z 535.0371) (a), UDP-Gal and UDP-Glc (m/z 565.0477) (b), and UDP-GalA and UDP-GlcA (m/z 579.0270) (c). Chromatographic and column regeneration conditions are given in the materials and methods section. EICCs were extracted with a ± 10 ppm mass window., sample, UDP-sugar standards (a-c) and UDP-sugars extracted from wild type Arabidopsis plants (d-f)

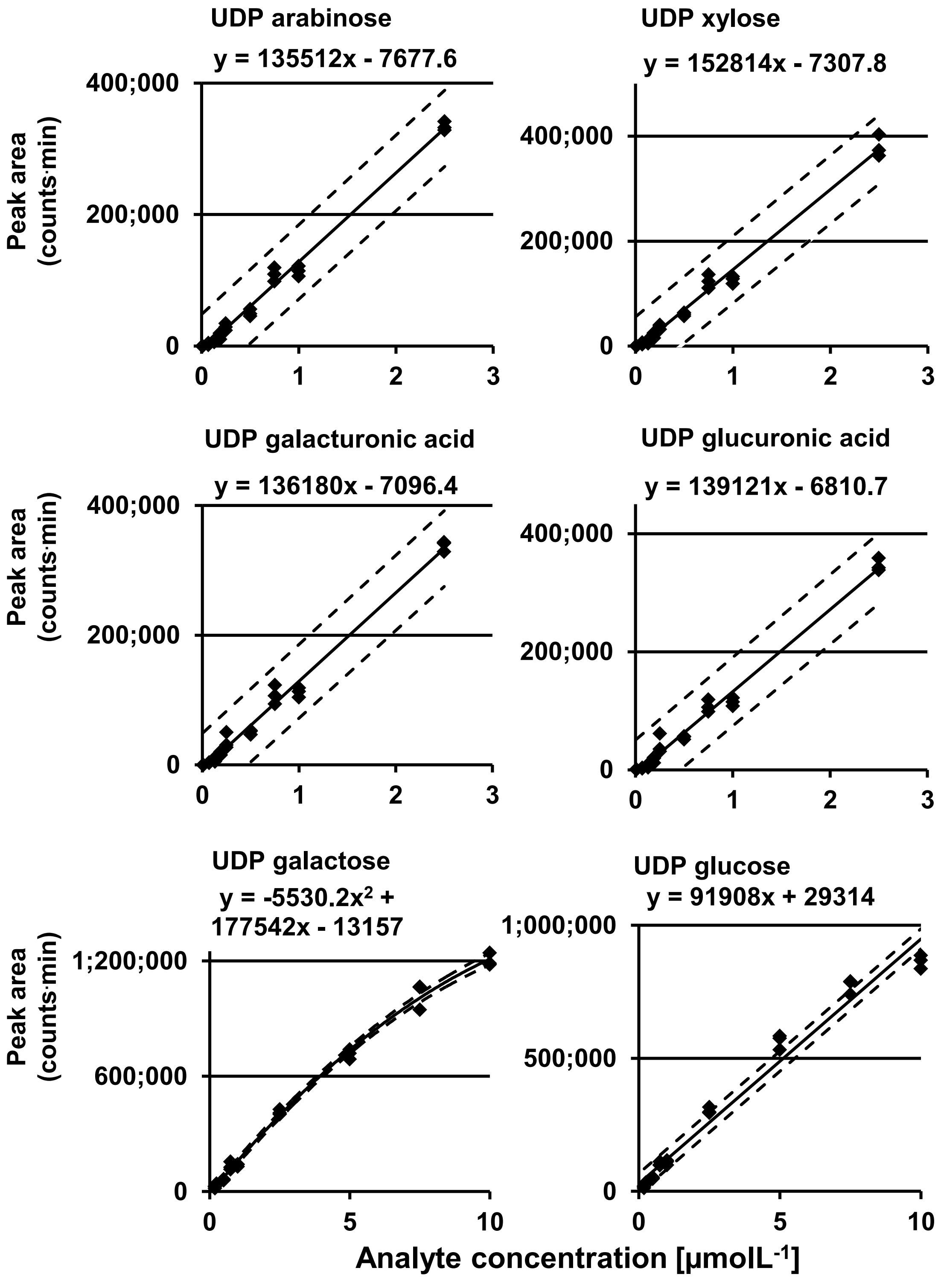

Fig. S2 Calibration graphs of six UDP-sugars. Parameters of the regression functions are given in Table 1

Table S1. Concentration of each UDP sugar and its confidence interval for each biological replicate[a)]

Wild type	Amount in µg g^{-1} fresh weight[a)]	C.I. in µg g^{-1} fresh weight[a)]	*ugd2,3* mutant	Amount in µg g^{-1} fresh weight[a)]	C.I. in µg g^{-1} fresh weight[a)]
UDP-Ara 1	0.84	0.28	UDP-Ara 1	0.44	0.28
UDP-Ara 2	0.76	0.28	UDP-Ara 2	0.60	0.28
UDP-Ara 3	0.60	0.28	UDP-Ara 3	0.46	0.28
UDP-Ara 4	1.14	0.28	UDP-Ara 4	0.56	0.28
UDP-Xyl 1	0.65	0.31	UDP-Xyl 1	0.38	0.31
UDP-Xyl 2	0.65	0.31	UDP-Xyl 2	0.52	0.31
UDP-Xyl 3	0.47	0.31	UDP-Xyl 3	0.38	0.31
UDP-Xyl 4	0.96	0.31	UDP-Xyl 4	0.42	0.31
UDP-Gal 1	3.22	0.71	UDP-Gal 1	5.49	0.75
UDP-Gal 2	3.58	0.72	UDP-Gal 2	5.88	0.76
UDP-Gal 3	2.79	0.70	UDP-Gal 3	5.20	0.75
UDP-Gal 4	5.47	0.75	UDP-Gal 4	6.50	0.78
UDP-Glc 1	22.69	2.24	UDP-Glc 1	32.24	2.30
UDP-Glc 2	26.22	2.25	UDP-Glc 2	36.27	2.37
UDP-Glc 3	19.35	2.23	UDP-Glc 3	33.03	2.31
UDP-Glc 4	37.79	2.40	UDP-Glc 4	38.52	2.42
UDP-GalA 1	0.64	0.34	UDP-GalA 1	0.32	0.34
UDP-GalA 2	0.58	0.34	UDP-GalA 2	0.46	0.34
UDP-GalA 3	0.41	0.34	UDP-GalA 3	0.41	0.34
UDP-GalA 4	0.75	0.34	UDP-GalA 4	0.41	0.34
UDP-GlcA 1	1.40	0.35	UDP-GlcA 1	0.50	0.35
UDP-GlcA 2	1.21	0.35	UDP-GlcA 2	0.56	0.35
UDP-GlcA 3	0.96	0.35	UDP-GlcA 3	0.47	0.35
UDP-GlcA 4	2.03	0.35	UDP-GlcA 4	0.55	0.35

[a)]Average concentrations and confidence intervals (N=3, P=95%) were calculated from triplicate measurements.

Table S2. Single factor ANOVA analysis results[a)]

SUMMARY for UDP arabinose

Groups	*Count*	*Sum*	*Average*	*Variance*
UDP-Ara WT	4	3.34	0.83	0.05
UDP-Ara *ugd2,3*	4	2.05	0.51	0.01

ANOVA

F	*F crit*	*P-value*
7.29	5.99	0.04

SUMMARY for UDP glucose

Groups	*Count*	*Sum*	*Average*	*Variance*
UDP-Glc WT	4	106.04	26.51	64.43
UDP-Glc ugd2,3	4	140.05	35.01	8.51

ANOVA

F	*F crit*	*P-value*
3.96	5.99	0.09

SUMMARY for UDP xylose

Groups	*Count*	*Sum*	*Average*	*Variance*
UDP-Xyl WT	4	2.73	0.68	0.04
UDP-Xyl *ugd2,3*	4	1.69	0.42	0.00

ANOVA

F	*F crit*	*P-value*
5.83	5.99	0.05

SUMMARY for UDP galacturonic acid

Groups	*Count*	*Sum*	*Average*	*Variance*
UDP-GalA WT	4	2.20	0.55	0.02
UDP-GalA *ugd2,3*	4	1.48	0.37	0.00

ANOVA

F	*F crit*	*P-value*
6.42	5.99	0.04

SUMMARY for UDP galactose

Groups	*Count*	*Sum*	*Average*	*Variance*
UDP-Gal WT	4	15.07	3.77	1.40
UDP-Gal *ugd2,3*	4	23.08	5.77	0.32

ANOVA

F	*F crit*	*P-value*
9.35	5.99	0.02

SUMMARY for UDP glucuronic acid

Groups	*Count*	*Sum*	*Average*	*Variance*
UDP-GlcA WT	4	5.17	1.29	0.18
UDP-GlcA *ugd2,3*	4	1.93	0.48	0.00

ANOVA

F	*F crit*	*P-value*
14.62	5.99	0.01

[a)] Wild type and *ugd2,3* data for each UDP sugar were compared for the four biological replicates via ANOVA analysis. The ANOVA analysis was performed by means of Microsoft Excel using an alpha value of 0.05. The F values are highlighted in red.
